# Interactions of marine sulfated glycans with antithrombin and platelet factor 4

**DOI:** 10.3389/fmolb.2022.954752

**Published:** 2022-09-19

**Authors:** Wenjing Zhang, Weihua Jin, Vitor H. Pomin, Fuming Zhang, Robert J. Linhardt

**Affiliations:** ^1^ Department of Endocrinology, Sir Run Run Shaw Hospital, Zhejiang University School of Medicine, Hangzhou, China; ^2^ Department of Chemical and Biological Engineering, Center for Biotechnology and Interdisciplinary Studies, Rensselaer Polytechnic Institute, Troy, NY, United States; ^3^ College of Biotechnology and Bioengineering, Zhejiang University of Technology, Hangzhou, China; ^4^ Department of BioMolecular Sciences, The University of Mississippi, Oxford, MS, United States; ^5^ Departments of Biological Science, Chemistry and Chemical Biology and Biomedical Engineering, Center for Biotechnology and Interdisciplinary Studies, Rensselaer Polytechnic Institute, Troy, NY, United States

**Keywords:** antithrombin, carbohydrate-protein interactions, heparin, platelet factor 4, sulfated glycans, surface plasmon resonance

## Abstract

The molecular interactions of sulfated glycans, such as heparin, with antithrombin (AT) and platelet factor 4 (PF4) are essential for certain biological events such as anticoagulation and heparin induced thrombocytopenia (HIT). In this study, a library including 84 sulfated glycans (polymers and oligomers) extracted from marine algae along with several animal-originated polysaccharides were subjected to a structure-activity relationship (SAR) study regarding their specific molecular interactions with AT and PF4 using surface plasmon resonance. In this SAR study, multiple characteristics were considered including different algal species, different methods of extraction, molecular weight, monosaccharide composition, sulfate content and pattern and branching vs. linear chains. These factors were found to influence the binding affinity of the studied glycans with AT. Many polysaccharides showed stronger binding than the low molecular weight heparin (e.g., enoxaparin). Fourteen polysaccharides with strong AT-binding affinities were selected to further investigate their binding affinity with PF4. Eleven of these polysaccharides showed strong binding to PF4. It was observed that the types of monosaccharides, molecular weight and branching are not very essential particularly when these polysaccharides are oversulfated. The sulfation levels and sulfation patterns are, on the other hand, the primary contribution to strong AT and PF4 interaction.

## Introduction

Antithrombin (AT) regulates the proteolytic activity of procoagulant proteases in both the intrinsic and extrinsic coagulation pathways and cause the anti-inflammatory signaling response through binding with syndecans-4 heparan sulfate proteoglycans (HSPGs) of different vascular endothelial cells (Carrell et al., 1997; Olson, Richard et al., 2010; Rezaie and Giri, 2020). Moreover, HSPGs with low affinity for AT stimulate potent proapoptotic and antiangiogenic activities (Rezaie and Giri, 2020).

Heparin induced thrombocytopenia (HIT) is a severe fatal immunothrombotic disorder resulting from the clinical application of heparin. Platelet factor 4 (PF4) is associated with HIT through a pathway involving the initial binding of PF4 to heparin to form heparin-PF4 neoepitope. This is followed by the production of anti-PF4-heparin antibodies and immunoglobulin G (IgG)-heparin-PF4 immune complex cluster, and finally platelet activation and aggregation (Zhang, Datta, Dordick, and Linhardt, 2020). The results of fluorescence spectroscopy indicated that two molecules of PF4 can bind to one molecule of high molecular weight (HMW) heparin and its avidity for HMW highly active heparin appeared to be at least 10- to 100- times greater than antithrombin’s avidity (Jordan, Favreau, Braswell and Rosenberg, 1982). Moreover, the carboxy-terminal amino acids of PF4 are critically important for binding to natural and synthetic GAGs (Loscalzo, Melnick, and Handin, 1985).

Heparin is a linear sulfated glycosaminoglycan, consisting mainly of disaccharide units 1→4 linked of *N*-sulfo, 6-*O*-sulfo *α*-D-glucosamine (GlcNS6S) and 1→4 linked 2-*O*-sulfo *α*-L-iduronic acid (IdoA2S) units. These trisulfated disaccharides can be accompanied with small amounts of *N*-acetyl *α*-D-glucosamine (GlcNAc) or *N*-sulfo, 3-*O*-sulfo, 6-*O*-sulfo *α*-D-glucosamine (GlcNS3S6S) disaccharide units. A specific pentasaccharide sequence (GlcNAc/NS6S-GlcA-GlcNS3S6S-IdoA2S-GlcNS6S) binds and activates AT resulting in heparin’s anticoagulant activity. The first trisaccharide unit (GlcNAc/NS6S-GlcA-GlcNS3S6S) is considered the initiator in the recognition of polysaccharide by the protein (Desai et al., 1998; Elli et al., 2020).

Other sulfated polysaccharides, as potential heparin mimetics, can be obtained in abundance from marine sources such as seaweeds. Seaweeds are taxonomically classified into three groups, green algae, red algae and brown algae. Polysaccharides from green algae contain two major types of sulfated polysaccharides, sulfated xylo-galacto-arabinans and sulfated xylo-glucurono-rhamnans (Jin, Zhang, Liang, and Zhang, 2016). Red algae have three major types of polysaccharides, the neutral polysaccharide agar, sulfated polysaccharide carrageenan and an agar-carrageenan hybrid. Brown algae contain the neutral polysaccharide laminaran, alginate and fucoidan. Fucoidans, a family of heteropolysaccharides, mainly consist of sulfated fucan, sulfated galactofucan and sulfated glucuronomannan derivatives (Deniaud-Bouet, Hardouin, Potin, Kloareg, and Herve, 2017). However, not all sulfated polysaccharides, like heparin, exhibit anticoagulant and/or antithrombotic activity (Pomin, 2014a, 2014b, 2015; Pomin and Mourão, 2014; Vasconcelos et al., 2018; Pomin et al., 2019). Fucosylated chondroitin sulfate has strong anticoagulant and antithrombotic activity, and the fucose pyranose branch and sulfation patterns are critical for this activity (Liu, Zhang, and Linhardt, 2009; Chen et al., 2013). A 2-*O*-sulfated fucose residue adversely impacts activity while 2-*O*-sulfated galactose and 4-*O*-sulfated fucose residues increase anticoagulation (Vasconcelos et al., 2018). Moreover, a high molecular weight is important for anticoagulation activity.

The anticoagulant activities of marine sulfated polysaccharides mainly result from their interaction with natural plasma serine proteases inhibitors (serpins), such as AT and heparin cofactor II (HC II) (Pomin, 2012). Marine sulfated polysaccharides inhibit coagulation through two distinct mechanisms. The first relies on the allosteric effects of serpins, particularly for HC II, and the second happens through a template mechanism, in which marine sulfated polysaccharides act as a “molecular bridge,” bringing serine protease and serpin together (Pomin, 2012).

Several series of oligosaccharides, polysaccharides and their derivatives were prepared in this work to find the candidates for antithrombotic treatments and antidotes to low molecular heparins. The interaction of marine algae polysaccharide with AT and PF4 were determined using surface plasmon resonance (SPR). The results obtained here were used to systematically analyze the structure-activity relationship found in the resultant intermolecular complexes made between marine sulfated glycans and AT/PF4, to identify specific characteristics that will contribute to the best molecular interactions.

## Materials and methods

### Materials

Heparin was purchased from Celsus Laboratories (Cincinnati, OH). Enoxaparin from Sandoz was generously provided by Dr. Jawed Fareed in Loyala University Medical Center (Maywood, IL). AT and human PF4 were purchased from Hyphen BioMed (Neuville-sur-Oise, France). Streptavidin (SA) sensor chips and HBS-EP buffer were purchased from Cytiva.

### Preparation of oligosaccharides, polysaccharides and their derivatives

**Scheme 1:**
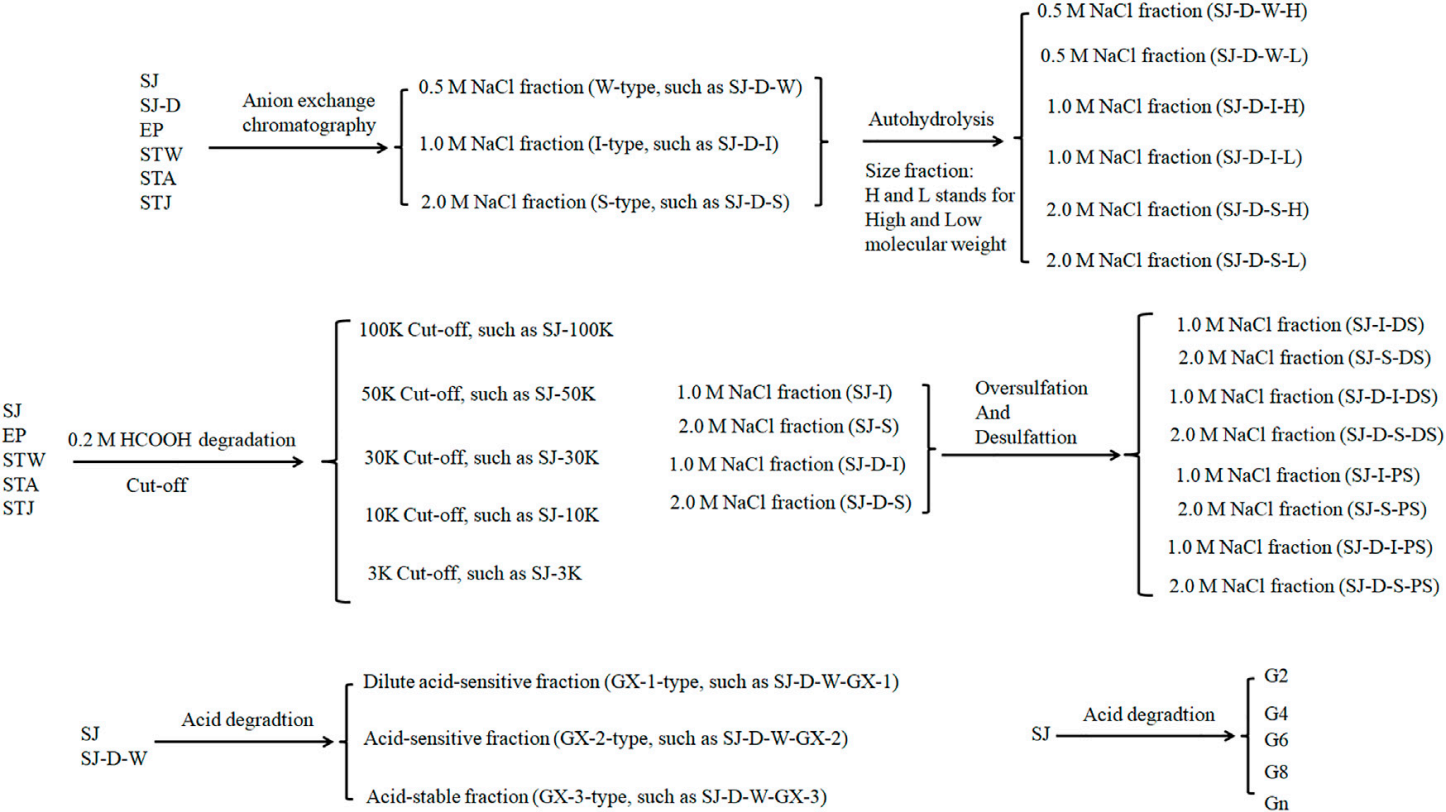
The flow chart of sulfated glycans preparation.

**FIGURE 1:**
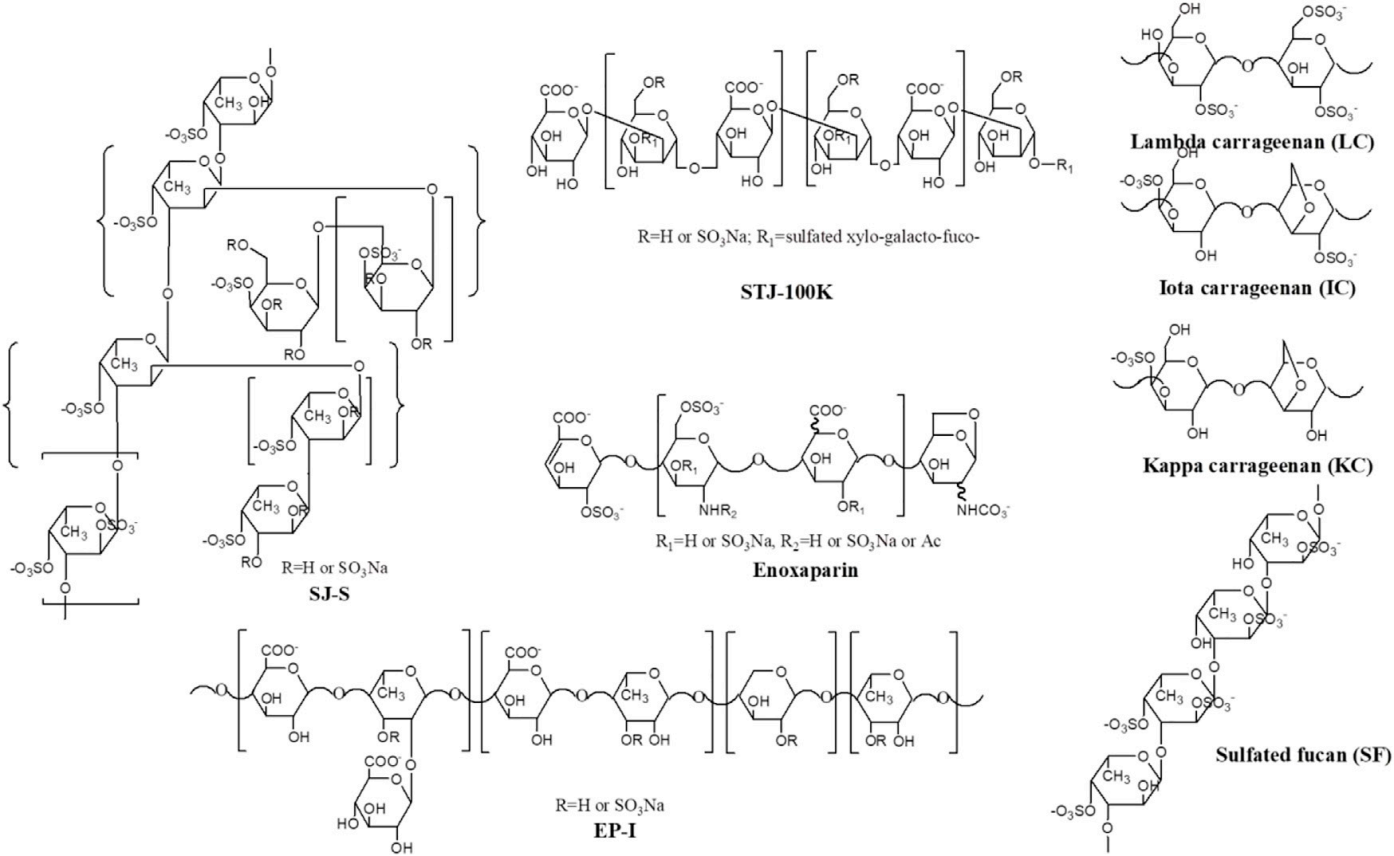
Proposed primary structures of polysaccharides used in this study.

Marine algae oligosaccharides, polysaccharides and their derivatives together with several animal-originated polysaccharides were prepared based on the previous studies (Ciancia et al., 2005; Wang et al., 2010; Anastyuk et al., 2012; Jin et al., 2012, Jin et al., 2013, Jin et al., 2017, Jin et al., 2018a, Jin et al., 2018b, Jin et al., 2019, Jin et al., 2020a, Jin et al., 2020b, Jin et al., 2020c, Jin et al., 2020d; Rodriguez-Jasso et al., 2013; Menshova et al., 2015; Shevchenko et al., 2015; Wu et al., 2015). They were summarized in Scheme 1, Table 1 with proposed structures in Figure 1 and more details can be found in Supplementary Table S1(Supplementary data 1).

**TABLE 1:** Samples and their abbreviation in this study.

NO	Abbreviation	Structural type	Characteristics
1	SJ	Fucoidan from *Saccharina japonica*	Crude polysaccharide obtained by hot water extraction
2	SJ-W	heteropolysaccharides	0.5 M NaCl fraction from SJ using anion exchange chromatography
3	SJ-I	Sulfated galactofucan	1.0 M NaCl fraction from SJ using anion exchange chromatography
4	SJ-S	Sulfated galactofucan	2.0 M NaCl fraction from SJ using anion exchange chromatography
5	SJ-D	Low molecular weight Fucoidan	Degraded from SJ
6	SJ-D-W	heteropolysaccharides	0.5 M NaCl fraction from SJ-D using anion exchange chromatography
7	SJ-D-I	Sulfated galactofucan	1.0 M NaCl fraction from SJ-D using anion exchange chromatography
8	SJ-D-S	Sulfated galactofucan	2.0 M NaCl fraction from SJ-D using anion exchange chromatography
9	SJ-D-W-H	heteropolysaccharides	High molecular weight fraction obtained by autohydrolysis from SJ-D-W
10	SJ-D-W-L	heteropolysaccharides	Low molecular weight fraction obtained by autohydrolysis from SJ-D-W
11	SJ-D-I-H	Sulfated galactofucan	High molecular weight fraction obtained by autohydrolysis from SJ-D-I
12	SJ-D-I-L	Sulfated galactofucan	Low molecular weight fraction obtained by autohydrolysis from SJ-D-I
13	SJ-D-S-H	Sulfated galactofucan	High molecular weight fraction obtained by autohydrolysis from SJ-D-S
14	SJ-D-S-L	Sulfated galactofucan	Low molecular weight fraction obtained by autohydrolysis from SJ-D-S
15	SJ-I-PS	Sulfated galactofucan	Oversulfation of SJ-I
16	SJ-S-PS	Sulfated galactofucan	Oversulfation of SJ-S
17	SJ-D-I-PS	Sulfated galactofucan	Oversulfation of SJ-D-I
18	SJ-D-S-PS	Sulfated galactofucan	Oversulfation of SJ-D-S
19	SJ-I-DS	Sulfated galactofucan	Desulfation of SJ-I
20	SJ-S-DS	Sulfated galactofucan	Desulfation of SJ-S
21	SJ-D-I-S	Sulfated galactofucan	Desulfation of SJ-D-I
22	SJ-D-S-DS	Sulfated galactofucan	Desulfation of SJ-D-S
23	SJ-100K	Fucoidan	Fraction obtained by using 100 K cut-off from 0.2 M HCOOH degradation of SJ
24	SJ-50K	Fucoidan	Fraction obtained by using 50 K cut-off from 0.2 M HCOOH degradation of SJ
25	SJ-30K	Fucoidan	Fraction obtained by using 30 K cut-off from 0.2 M HCOOH degradation of SJ
26	SJ-10K	Fucoidan	Fraction obtained by using 10 K cut-off from 0.2 M HCOOH degradation of SJ
27	SJ-3K	Fucoidan	Fraction obtained by using 3 K cut-off from 0.2 M HCOOH degradation of SJ
28	SJ-GX-1	Fucoidan	Dilute acid-sensitive fraction obtained by acid degradation of SJ
29	SJ-GX-2	Fucoidan	Acid-sensitive fraction obtained by acid degradation of SJ
30	SJ-GX-3	Fucoidan	Acid-stable fraction obtained by acid degradation of SJ
31	SJ-D-W-GX-1	Fucoidan	Dilute acid-sensitive fraction obtained by acid degradation of SJ-D-W
32	SJ-D-W-GX-2	Fucoidan	Acid-sensitive fraction obtained by acid degradation of SJ-D-W
33	SJ-D-W-GX-3	Fucoidan	Acid-stable fraction obtained by acid degradation of SJ-D-W
34	G2	Glucuronomannan-dimer	β-D-glucuronosyluronic acid-(1→2)-α/β-D-mannose
35	G4	Glucuronomannan-tetramer	β-D-glucuronosyluronic acid-(1→2)-α-D-mannose-(1→4) -β-D-glucuronosyluronic acid-(1→2)-α/β-D-mannose
36	G6	Glucuronomannan-hexamer	β-D-glucuronosyluronic acid-(1→2)-α-D-mannose-(1→4) -β-D-glucuronosyluronic acid-(1→2)-α-D-mannose-(1→4) -β-D-glucuronosyluronic acid-(1→2)-α/β-D-mannose
37	Gn	glucuronomannan	Poly (β-D-glucuronosyluronic acid-(1→2)-α-D-mannose-(1→4), alternating)
38	STW	Fucoidan from *Sargassum thunbergii*	Crude polysaccharide obtained by hot water extraction
39	STA	Fucoidan from *Sargassum thunbergii*	Crude polysaccharide obtained by acid extraction
40	STJ	Fucoidan from *Sargassum thunbergii*	Crude polysaccharide obtained by alkali extraction
41	EP	sulfated glucurono-xylo-rhamnan	Crude polysaccharide obtained by hot water extraction
42	STW-W	heteropolysaccharides	0.5 M NaCl fraction from STW using anion exchange chromatography
43	STW-I	Sulfated galactofucan (Mainly)	1.0 M NaCl fraction from STW using anion exchange chromatography
44	STW-S	Sulfated galactofucan	2.0 M NaCl fraction from STW using anion exchange chromatography
45	STA-W	heteropolysaccharides	0.5 M NaCl fraction from STA using anion exchange chromatography
46	STA-I	Sulfated galactofucan (Mainly)	1.0 M NaCl fraction from STA using anion exchange chromatography
47	STA-S	Sulfated galactofucan	2.0 M NaCl fraction from STA using anion exchange chromatography
48	STJ-W	heteropolysaccharides	0.5 M NaCl fraction from STJ using anion exchange chromatography
49	STJ-I	Sulfated galactofucan	1.0 M NaCl fraction from STJ using anion exchange chromatography
50	STJ-S	Sulfated galactofucan	2.0 M NaCl fraction from STJ using anion exchange chromatography
51	EP-W	sulfated glucurono-xylo-rhamnan	0.3 M NaCl fraction from EP using anion exchange chromatography
52	EP-I	sulfated glucurono-xylo-rhamnan	1.0 M NaCl fraction from EP using anion exchange chromatography
53	EP-S	sulfated glucurono-xylo-rhamnan	2.0 M NaCl fraction from EP using anion exchange chromatography
54	STW-100K	Fucoidan	Fraction obtained by using 100 K cut-off from 0.2 M HCOOH degradation of STW
55	STW-50K	Fucoidan	Fraction obtained by using 50 K cut-off from 0.2 M HCOOH degradation of STW
56	STW-30K	Fucoidan	Fraction obtained by using 30 K cut-off from 0.2 M HCOOH degradation of STW
57	STW-10K	Fucoidan	Fraction obtained by using 10 K cut-off from 0.2 M HCOOH degradation of STW
58	STW-3K	Fucoidan	Fraction obtained by using 3 K cut-off from 0.2 M HCOOH degradation of STW
59	STA-100K	Fucoidan	Fraction obtained by using 100 K cut-off from 0.2 M HCOOH degradation of STA
60	STA-50K	Fucoidan	Fraction obtained by using 50 K cut-off from 0.2 M HCOOH degradation of STA
61	STA-30K	Fucoidan	Fraction obtained by using 30 K cut-off from 0.2 M HCOOH degradation of STA
62	STA-10K	Fucoidan	Fraction obtained by using 10 K cut-off from 0.2 M HCOOH degradation of STA
63	STA-3K	Fucoidan	Fraction obtained by using 3 K cut-off from 0.2 M HCOOH degradation of STA
64	STJ-100K	Fucoidan	Fraction obtained by using 100 K cut-off from 0.2 M HCOOH degradation of STJ
65	STJ-50K	Fucoidan	Fraction obtained by using 50 K cut-off from 0.2 M HCOOH degradation of STJ
66	STJ-30K	Fucoidan	Fraction obtained by using 30 K cut-off from 0.2 M HCOOH degradation of STJ
67	STJ-10K	Fucoidan	Fraction obtained by using 10 K cut-off from 0.2 M HCOOH degradation of STJ
68	STJ-3K	Fucoidan	Fraction obtained by using 3 K cut-off from 0.2 M HCOOH degradation of STJ
69	EP-100K	sulfated glucurono-xylo-rhamnan	Fraction obtained by using 100 K cut-off from 0.2 M HCOOH degradation of EP
70	EP-50K	sulfated glucurono-xylo-rhamnan	Fraction obtained by using 50 K cut-off from 0.2 M HCOOH degradation of EP
71	EP-30K	sulfated glucurono-xylo-rhamnan	Fraction obtained by using 30 K cut-off from 0.2 M HCOOH degradation of EP
72	EP-10K	sulfated glucurono-xylo-rhamnan	Fraction obtained by using 10 K cut-off from 0.2 M HCOOH degradation of EP
73	EP-3K	sulfated glucurono-xylo-rhamnan	Fraction obtained by using 3 K cut-off from 0.2 M HCOOH degradation of EP
74	Enoxaparin	Enoxaparin	Purchased from Teva Parenteral Medicines, Inc
75	LC	λ-carrageenan	Purchased from Millipore Sigma
76	IC	iota carrageenan	Purchased from Millipore Sigma
77	KC	kappa carrageenan	Purchased from Millipore Sigma
78	Agar	Agar	Purchased from Millipore Sigma
79	Des-H	Desulfated heparin	Provided by Robert J. Linhardt’s Lab
80	Heparosan	Heparosan	Provided by Robert J. Linhardt’s Lab
81	SF	Sulfated fucan	SF, provided by Vitor H Pomin, was extracted from *Lytechinus variegates* (sea urchin)
82	LA-PS	Sulfated laminaran	Oversulfation of laminaran
83	LA	Laminaran from *Sargassum thunbergii*	Water fraction from STW using anion exchange chromatography
84	LAO	Glucoglucuronan	Oxidized laminarin

### Surface plasmon resonance analysis

Biotinylated heparin was immobilized on a streptavidin (SA) chip to prepare a heparin chip (Zhang et al., 2019). In brief, 20 μl solution of biotinylated heparin (0.1 mg/ml) in HBS-EP + buffer (0.01 M 4-(2-hydroxyethyl)-1-piperazineethanesulfonic acid, 0.15 M NaCl, 3 mM EDTA, 0.05% surfactant P20, pH 7.4) was injected over flow cell 2 (FC2), 3 (FC3) and 4 (FC4) of the SA chips at a flow rate of 10 μl/min. The successful immobilization of GAGs was confirmed by the observation of a ∼200 resonance unit (RU) increase in the sensor chip. The control flow cell (FC1) was prepared by 1 min injection with saturated biotin.

Kinetic measurements of interactions between AT-heparin and PF4-heparin were performed. Briefly, 90 μl different concentrations of AT and PF4 diluted in HBS-EP buffer were injected over to the heparin chip at a flow rate of 30 μl/min. After each run, there was a 3 min dissociation time and a 1 min regeneration time using 2 M NaCl for AT and 10 mM glycin-HCl pH 2.5 buffer and 2 M NaCl for PF4. The response (RU) was monitored as a function of time (sensorgram) at 25°C.

Solution competition SPR was used to examine the relative binding affinity of sulfated glycans with AT or PF4. Protein (AT or PF4) was pre-mixed with different sulfated glycans before injection into the heparin chip. Once the active binding sites on AT or PF4 were occupied by glycan in solution, its binding to the surface-immobilized heparin decreased, resulting in a reduction of signal (RU) in a concentration-dependent fashion. Competition study was performed by injecting 90 μL mixtures of 1 µM glycans, including enoxaparin, with 250 nM of AT or 25 nM of PF4 for comparing the binding abilities of different sulfated glycans. For IC_50_ measurements, five different concentrations of glycan samples (from 0.5 to 1,000 nM), including enoxaparin, were premixed with 250 nM of AT or 25 nM of PF4, then injected to the heparin chip to test the inhibition on heparin-AT, or heparin-PF4 interaction. Other protocols were performed as described above.

## Results and discussion

### Kinetics measurements of antithrombin and PF4 interaction with heparin

**FIGURE 2:**
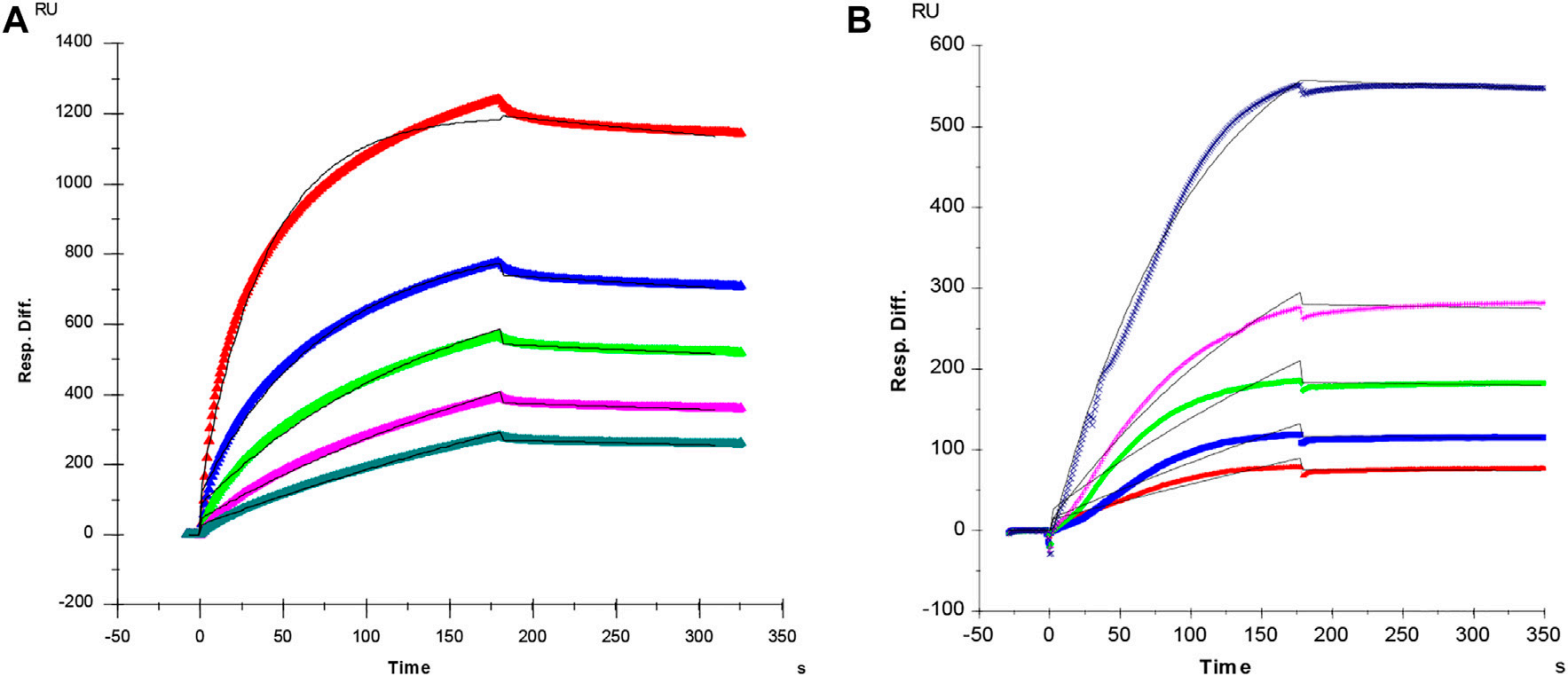
**(A)** SPR sensorgram of AT-heparin interaction. Concentrations of AT (from top to bottom) were 1,000, 500, 250, 125, and 63 nM, respectively. The AT-heparin-binding kinetics were determined by global fitting the curves to a 1:1 biomolecular reaction model (black lines) using the BIA evaluation software 4.01. **(B)** SPR sensorgram of PF4-heparin interaction. Concentrations of PF4 (from top to bottom) were 100, 50, 25, 12.5, and 6.3 nM, respectively. The PF4-heparin binding kinetics were determined by globally fitting the curves to a 1:1 biomolecular reaction model (black lines) using the BIAevaluation software 4.0.1.

It is well known that the molecular interactions of AT and PF4 with heparin are essential factors for heparin anticoagulant and heparin induced thrombocytopenia (HIT). In current study, SPR was applied to measure the binding kinetics and affinity of AT and PF4 interaction with heparin using a sensor chip with immobilized heparin. Sensorgrams of AT and PF4 interactions with heparin are shown in Figure 2. Binding kinetics (i.e., association rate constant: *ka*; dissociation rate constant: *kd*) and affinity (i.e., *K*_*D*_ *= kd/ka*) were calculated by globally fitting the sensorgrams using 1:1 Langmuir binding model. AT-heparin interaction: *ka* = 1.2 × 10^5^ 1/Ms, *kd* = 4.4 × 10^–4^ 1/s, and *K*_*D*_ = 3.6 nM; PF4-heparin interaction: *ka* = 9.6 × 10^4^ 1/Ms, *kd* = 1.1 × 10^–4^ 1/s, and *K*_*D*_ = 1.2 nM.

### Surface plasmon resonance competition study on polysaccharides fractionated by anion exchange chromatography on heparin chip binding to antithrombin

**FIGURE 3:**
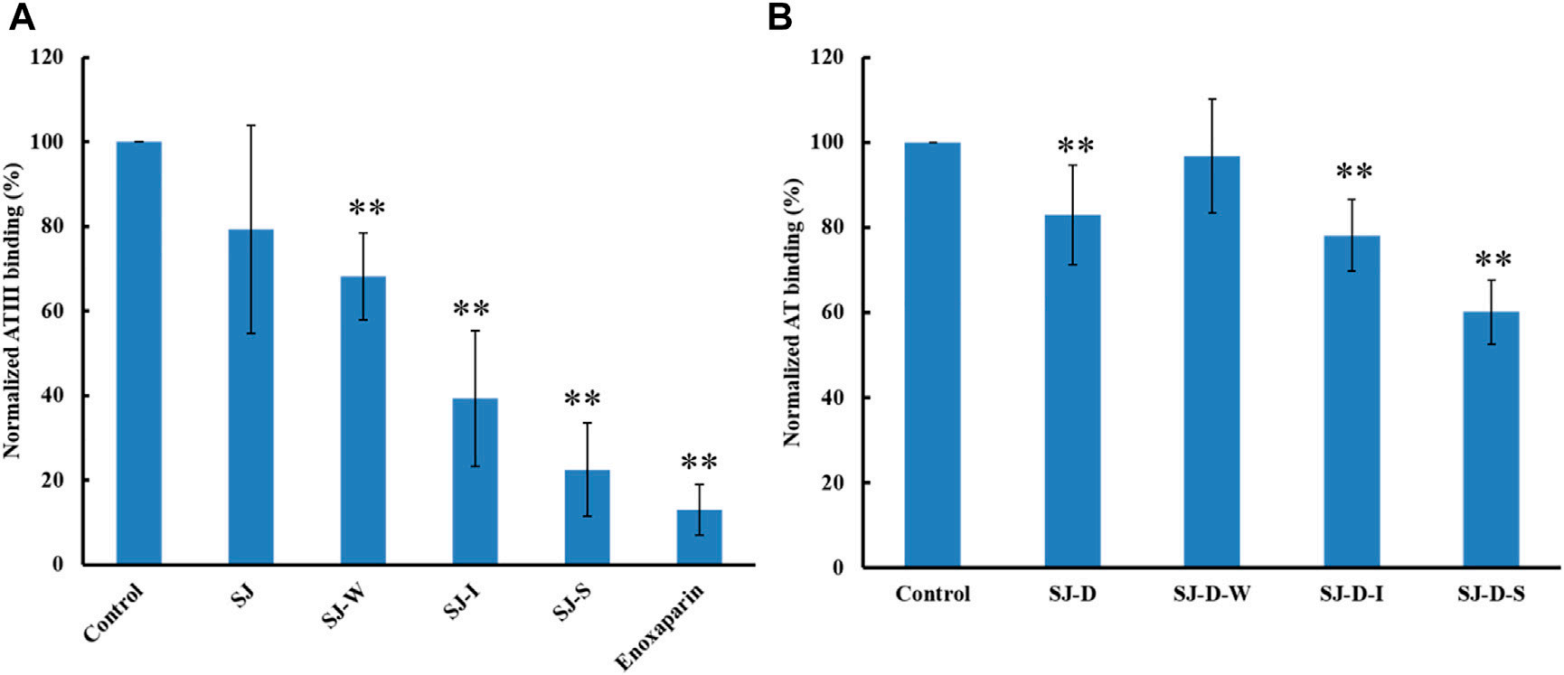
The binding abilities to AT (250 nM) premixed with different MW of polysaccharides (1 µM) on a heparin chip. **(A)** SJ and its fractions; **(B)** low molecular weight SJ-D and its fractions. All bar graphs with standard deviations were based on triplicate experiments. *p < 0.05, **p < 0.01 compared with control.

The relative binding affinities to AT of crude polysaccharide from brown algae *Saccharina japonica* (SJ) and its derivatives were determined by SPR. SJ-S is a sulfated galactofucan based on previous studies (Jin, et al., 2020d). SJ-S, having the highest negative charge, showed the strongest inhibition of binding (78% vs. 87% enoxaparin) (Figure 3A), indicating that SJ-S was an active component. SJ-D was prepared to evaluate the impact of molecular weight on AT interaction. The inhibitory activities of SJ and SJ-D were similar, suggesting that molecular weight of these two samples did not influence their competitive binding ability. In contrast, low molecular weight fractions (SJ-D-W, SJ-D-I, and SJ-D-S) showed lower competitive binding to AT (Figure 3B) than the corresponding higher molecular weight fractions, suggesting that molecular weight is important factor to bind AT.

### Competition study on modified polysaccharides (mainly sulfated galactofucans) on heparin chip binding to antithrombin

**FIGURE 4:**
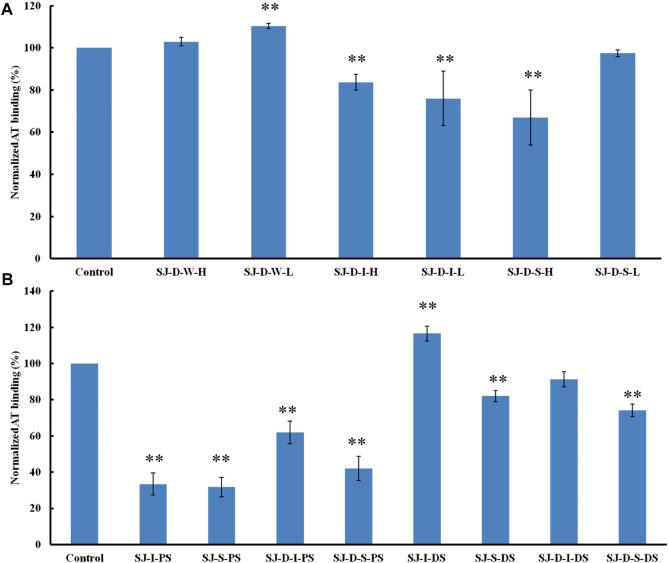
The binding abilities to AT (250 nM) premixed with different chemical modified polysaccharides (1 µM) on a heparin chip. **(A)** Polysaccharides obtained from autohydrolysis and **(B)** desulfated or oversulfated polysaccharides. All bar graphs with standard deviations were based on triplicate experiments. *p < 0.05, **p < 0.01 compared with control.

The AT-binding polysaccharides are sulfated galactofucan based on the results of heparin competition studies. Autohydrolysis, desulfation and oversulfation were performed to study the effects of sulfation level, sulfation pattern and molecular weight. The H-type fractions, obtained by autohydrolysis, display similar inhibitory activities as the corresponding unfractionated material (Figure 4A). C2-selective desulfation of fucopyranose (Fuc) and degradation happen during the process of autohydrolysis (Ciancia et al., 2005; Pomin et al., 2005; Anastyuk et al., 2012; Rodriguez-Jasso et al., 2013; Menshova et al., 2015; Shevchenko et al., 2015; Wu et al., 2015). This suggests that sulfation at the C2 of Fuc residues is not crucial for AT-binding. Desulfation lowered AT-binding while oversulfation increased AT-binding of low molecular weight fractions (SJ-D-I and SJ-D-S) (Figure 4B). Oversulfation of SJ-I and SJ-S had little impact on AT-binding. These results indicate that molecular weight and sulfation are necessary for the AT-binding of sulfated galactofucans.

### Competition study on modified polysaccharides (glucuronomannan derivatives) on heparin chip binding to antithrombin

**FIGURE 5:**
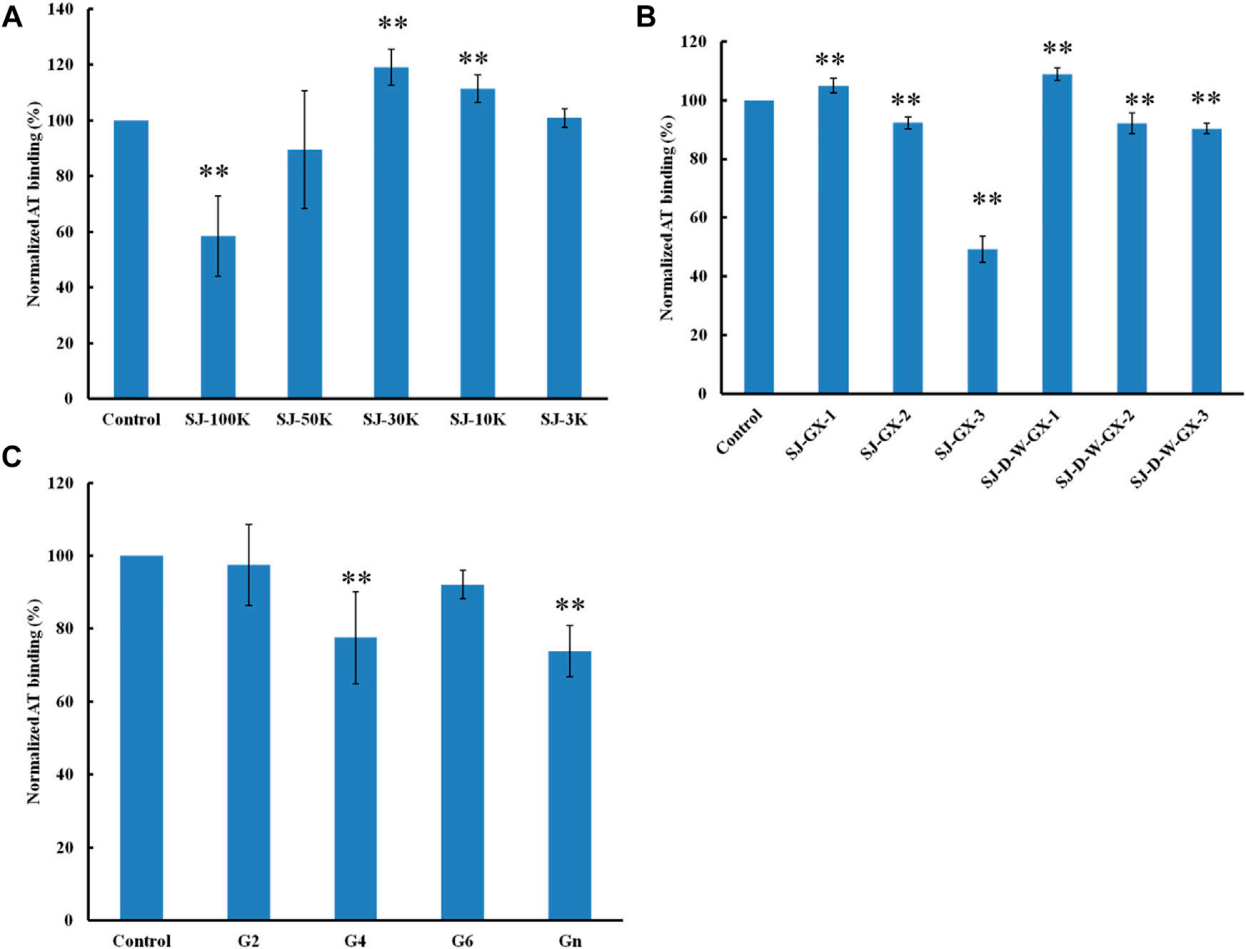
The binding abilities to AT (250 nM) premixed with different glycans (1 µM) on a heparin chip. **(A)** glycans derived from MWCO membranes; **(B)** fractions obtained from acid degradation; **(C)** glucuronomannan (Gn) and its oligomers. All the bar graphs with standard deviations were based on triplicate experiments. *p < 0.05, **p < 0.01 compared with control.

Formic acid (0.2 M) was used to degrade SJ and separated by five molecular weight cut off (MWCO) membranes (100, 50, 30, 10 and 3 K). The larger molecular weight fractions showed stronger binding to AT, suggesting that acid stable fractions might show the strongest binding to AT (Figure 5). According to previous studies (Jin et al., 2012; Wu et al., 2015; Deniaud-Bouet et al., 2017), acid stable fractions were glucuronomannan derivatives. SJ-GX-3 displayed stronger inhibition of binding than SJ-GX-1 and SJ-GX-2, which confirmed that the acid stable fractions showed the best competition for heparin binding to AT. However, it was disappointing that the glucuronomannan (Gn) with 7.0 kDa showed low AT-binding, which might be best based on its molecular weight. In summary, we hypothesize that polysaccharides that elute from ion-exchange resin with 2 M NaCl or are retained with 100 kDa cut-off exhibit the strongest binding to AT.

### Competition study on different polysaccharides on heparin chip binding to antithrombin

**FIGURE 6:**
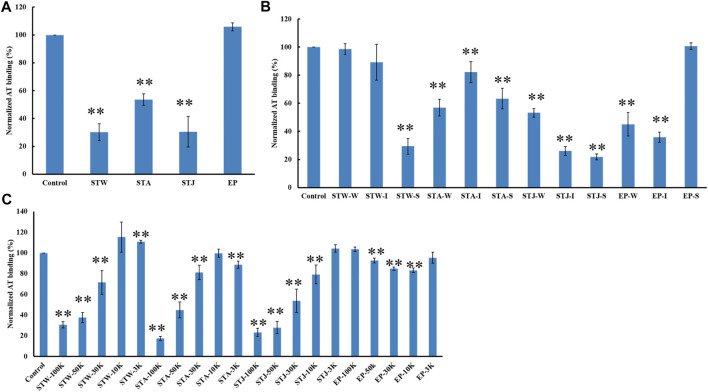
The binding abilities of AT (250 nM) premixed with different polysaccharides on a heparin chip. **(A)** Crude polysaccharides; **(B)** fractions obtained by anion exchange chromatography; **(C)** fractions derived from MWCO membranes. All bar graphs with standard deviations were based on triplicate experiments. *p < 0.05, **p < 0.01 compared with control.

The AT-binding affinity of STW (70%) was like STJ’s (70%) and larger than STA’s (46%) (Figure 6A). These results can be explained by degradation and desulfation that occurs under conditions of acidic extraction. EP showed no AT-binding, suggesting that the extraction methods and different algae sulfated polysaccharides impacted binding.

STW, STA, STJ, and EP were separated by anion-exchange chromatography and MWCO membranes to confirm the impact of size and change on AT-binding. Thirty-two fractions were obtained and determined the binding ability to bind to AT. STW-S (71%) had the strongest inhibition of binding, followed by STW-I and STW-W showed the weakest inhibition of binding (Figure 6B). Fractions of STJ showed similar pattern, which is STJ-S (78%) > STJ-I > STJ-W. However, the AT-binding affinity of STJ’s fractions and EP’s fractions showed different patterns, which were EP-I > EP-W > EP-S ≈ EP and STA ≈ STA-W > STA-S > STA-I, respectively.

We suggest that while elution from an anion exchange resin with 2 M NaCl, corresponds to the fraction with the highest charge, this fraction does not always exhibit the strongest AT-binding affinity. The AT-binding affinity of fractions obtained by MWCO membranes was next determined (Figure 6C). Components retained with 100 kDa cut-off exhibited the strongest binding to AT except for the EP fractions. This might be explained by the different behavior of different types of polysaccharides. Polysaccharides from *S. japonica* and *S. thunbergii* belonged to fucose-containing sulfated polysaccharides (FCSPs) while polysaccharides from *E. prolifera* belong to rhamnose-containing sulfated polysaccharides. These data show that FCSPs components retained with 100 kDa cut-off showed the strongest AT-binding.

### IC_50_ measurements of polysaccharides inhibiting antithrombin interaction with heparin on chip

Based on the above results, we propose that fractions eluting with 2 M NaCl and retained with 100 and 50 kDa cut-off showed the strongest AT-binding. Therefore, we determined the 50% inhibition concentration (IC_50_) of five S-type fractions, 100K-type fractions and 50K-type fractions (Table 2). The IC_50_ of the fractions derived from STA were larger than fractions obtained from STJ and STW, suggesting that acid-treated decrease AT-binding activity. In addition, polysaccharides from different algae had different acid-sensitivity, which was confirmed by the comparison of SJ-100K and STW-100K or SJ-50K and STW-50K fractions.

**TABLE 2:** IC_50_ values of sulfated glycans on inhibiting AT binding to heparin (on chip surface).

Samples	IC50 (nM)	Samples	IC50 (nM)	Samples	IC50 (nM)
SJ-S	9	SJ-100K	2,948	SJ-50K	4,314
STW-S	103	STW-100K	47	STW-50K	89
STA-S	740	STA-100K	117	STA-50K	555
STJ-S	41	STJ-100K	37	STJ-50K	119
EP-S	>1,000	EP-100K	>1,000	EP-50K	>1,000
EP-I	134	SJ-GX-3	1,000	SJ-D-S	5,675
SF	1840	LA-PS	86	LC	9
Enoxaparin	444				

**TABLE 3:** IC_50_ values of sulfated glycans on inhibiting PF4 binding to heparin (on chip surface).

Samples	IC_50_ (nM)	Samples	IC_50_ (nM)	Samples	IC_50_ (nM)
STW-S	4.0	STW-100K	10.8	STW-50K	16.3
STA-S	1.1	STA-100K	4.8	STA-50K	27.1
STJ-S	1.5	STJ-100K	2.5	STJ-50K	10.4
SJ-S	1.0	EP-I	>1,000	LC	2.3
Enoxaparin	12.6	SF	1.0	LA-PS	1.8

Higher molecular weight fractions showed lower IC_50_ values, which was also consistent with results for SJ-D-S (The IC_50_ was 5.7 μM). It is interesting to note that the IC_50_ of EP-S, EP-100K and EP-50K were ≥10 μM while the IC_50_ of EP-I was 134 nM value. There are 11 sulfated glycans having lower IC_50_ values in Table 2 than that for the low molecular weight heparin, enoxaparin (∼444 nM). Among these, the IC_50_ values of SJ-S, STJ-S, STW-100K, STJ-100K, LA-PS, STW-50K and LC were below 100 nM. The kinetic measurements of the interaction of polysaccharides with AT were determined by solution-based affinities (Ki), using the equation: Ki = IC_50_/(1 + [C]/K_D_), where [C] is the concentration of AT (250 nM) used in the competition SPR and the dissociation constant (K_D_) for heparin and AT was 151 nM (Zhao, Kong, Zhang, and Linhardt, 2018; Jin, et al., 2020c). It was found that the smallest Ki of three were SJ-S (3 nM), STJ-100K (14 nM) and STJ-S (15 nM). The Ki of EP-I was 50 nM.

**FIGURE 7:**
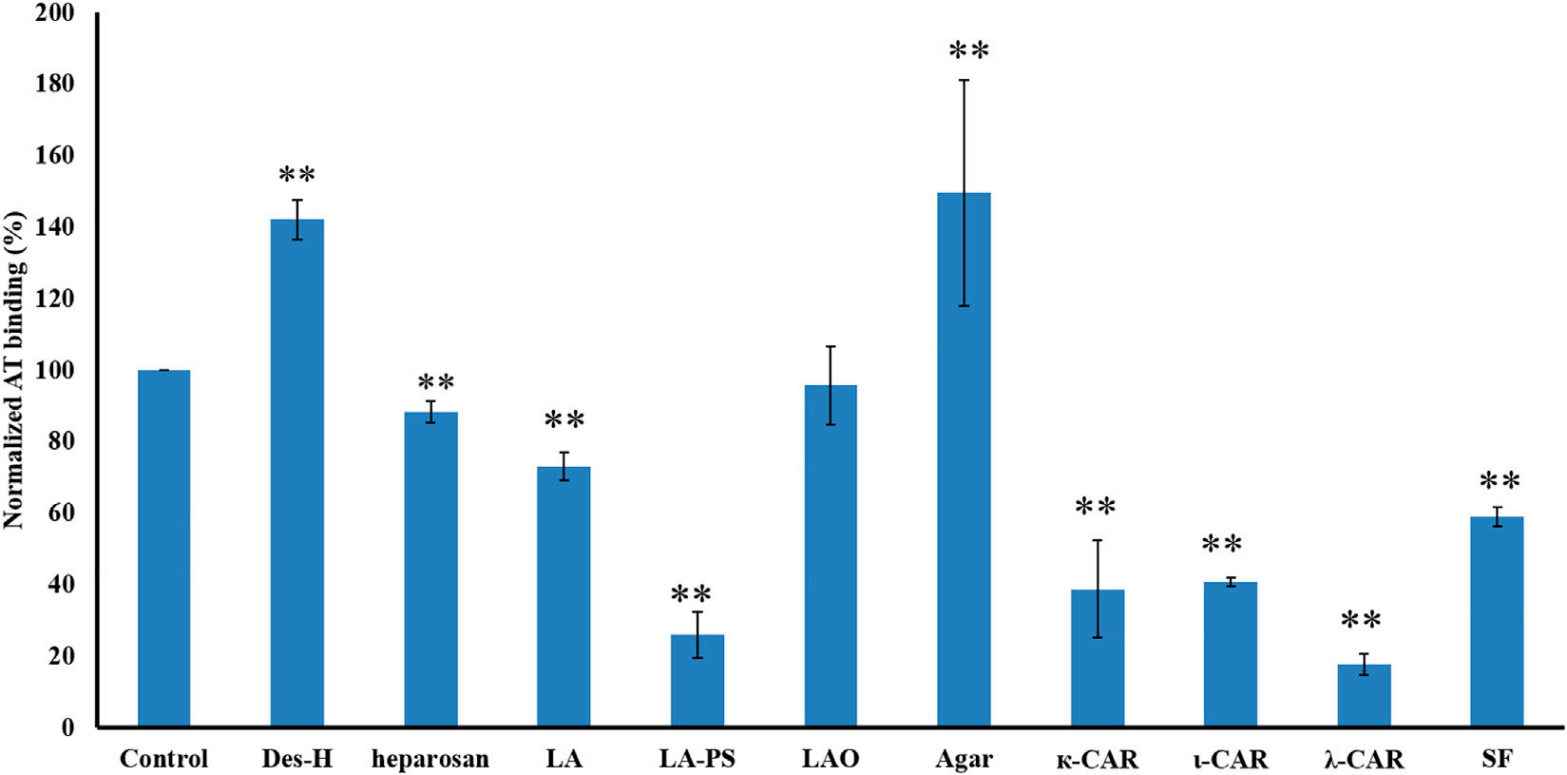
The binding abilities to AT of different polysaccharides on a surface heparin chip. Concentrations of AT and sulfated glycan samples were 250 nM and 1 μM, respectively. All bars with standard deviations were based on triplicate experiments. *p < 0.05, **p < 0.01 compared with control.

SJ-S was a sulfated galactofucan, STJ-100K was a sulfated glucuronomannan derivative and EP-I was a sulfated xylo-glucuronorhamnan (Figure 1). By examining heparin, we found that sulfation was required as desulfated heparin (Des-H) and the heparin precursor, (heparosan), showed no AT-binding (Figure 7). In addition, we found that the type of monosaccharides comprising the polysaccharide were unimportant for AT-binding, which was also confirmed by the result obtained over sulfated laminarin (LA-PS) (Figure 7). It is harder to draw a firm conclusion about the importance of glucuronic acid (GlcA) residues for AT-binding. Oxidized laminarin (LAO) showed a weak inhibition of binding (Figure 7). Therefore, GlcA might not be essential, particularly if most GlcA residues were are sulfated. It has been reported that the critical glucuronic acid in the first trisaccharides of the AT-binding site can be replaced by 2-*O*-sulfated iduronic acid (Elli et al., 2020).

The binding abilities of five types of sulfated galactans, agar (containing no sulfation), kappa-carrageenan (one sulfate per disaccharide unit), iota-carrageenan (two sulfates per disaccharide unit) and lambda-carrageenan (three sulfates per disaccharide unit) were next determined. These data suggest that the types of monosaccharide residues present had no impact on AT-binding and that binding increased with the increasing content of sulfate. A linear sulfated fucan (2.5 sulfates per disaccharide unit, SF) was also used to determine the binding affinity. The IC_50_ of SF (1840 nM) was higher than SJ-S (9 nM), suggesting that branching was important.

### IC_50_ measurements of polysaccharides inhibiting PF4 interaction with heparin on chip

It is known that 0.2%–3% patients show HIT when treated with heparin, which can lead to life threatening thrombosis and clinical symptoms (Fischer, 2001). Therefore, it is important to understand the binding affinity of anticoagulants to PF4. The IC_50_ of 14 candidates in inhibiting PF4 binding to heparin were determined in Table 3. The IC_50_ of enoxaparin was 12.6 nM and only three candidates (EP-I, STW-50K and STA-50K) showed higher IC_50_. Compared 100K-type fractions with 50K-type fractions, it was concluded that low molecular weight fractions had lower binding abilities to PF4. It was noteworthy that EP-I exhibited the lowest binding affinity for among the candidates and the IC_50_ of EP-I was larger than 1 µM.

## Conclusion

A systematic study was performed to study the structure-activity relationship between marine algae sulfated polysaccharides and AT and PF4 binding to find the candidates for antithrombotic treatments and antidotes to low molecular heparins. Comparing SJ with its fractions, it was found that sulfated galactofucan showed the strongest inhibitory activity. The results on the binding abilities of samples with oversulfation, desulfation and desulfation at the C2 of Fuc residues suggested that the sulfation level and proper sulfation pattern are of primary importance on AT and PF4 binding in most cases. SJ and SJ-D had similar binding abilities to AT while SJ-100K, SJ-50K, SJ-30K, SJ-10K, and SJ-3K had different binding abilities, indicating that molecular weight may be not a major factor, which was confirmed by the results of other fractions derived from MWCO membranes. The AT-binding affinities of four crude polysacchairdes (STW, STA, STJ, and EP) showed that the extraction methods and different algae sulfated polysaccharides could impact the binding abilities. Simply binding to AT does necessarily mean that antithrombin becomes activated as a protease inhibitor. The target protease is thrombin (rather than Factor Xa) as previous studies (Pomin, 2012) demonstrated that sulfated marine glycans are primarily active as anticoagulants by direct binding to the serine protease thrombin or by indirectly binding to HC II, a serine protease inhibitor acting on thrombin. Sulfated laminaran, carrageenan, sulfated fucan, sulfated galactofucans, sulfated glucuronomannan derivatives and sulfated xylo-glucuronorhamnan showed strong inhibitory activities, suggesting that the types of monosaccharides present are unimportant, particularly when these polysaccharides are oversulfated. Finally, a linear sulfated fucan from (sea urchin) *Lytechinus variegates* and other branched sulfated galactofucans had similar IC_50_ values on inhibiting PF4 binding to heparin, indicating branching is not a major factor for polysaccharide binding.

## Data Availability

The datasets presented in this study can be found in online repositories. The names of the repository/repositories and accession number(s) can be found in the article/Supplementary Material.
