# Supplementary material for: Interactions of marine sulfated glycans with antithrombin and platelet factor 4
Source: Front Mol Biosci. 2022 Sep 19;9:954752. doi: 10.3389/fmolb.2022.954752 (PMC9527323; doi:10.3389/fmolb.2022.954752)
Supplement: Supplementary file 1 [file DataSheet1.docx]

**Interactions of marine sulfated glycans with antithrombin and platelet factor 4**

Wenjing Zhang^1^, Weihua Jin ^2, 3*^, Vitor H Pomin^4^, Fuming Zhang^2*^ and Robert J. Linhardt^2,5^^[[1]](#footnote-1)^*

^1^Department of Endocrinology, Sir Run Run Shaw Hospital, Zhejiang University School of Medicine, Hangzhou, China.

^2^Department of Chemical and Biological Engineering, Center for Biotechnology and Interdisciplinary Studies, Rensselaer Polytechnic Institute, Troy, NY, USA

^3^College of Biotechnology and Bioengineering, Zhejiang University of Technology, Hangzhou, China

^4^Department of BioMolecular Sciences, The University of Mississippi, MS, USA

^5^Departments of Biological Science, Chemistry and Chemical Biology and Biomedical Engineering, Center for Biotechnology and Interdisciplinary Studies, Rensselaer Polytechnic Institute, Troy, NY, USA

**Supplementary data**

1. **Preparation of oligosaccharides, polysaccharides and their derivatives**

Based on previous studies [1-5], we prepared six polysaccharides, crude polysaccharide from *Saccharina japonica* (brown algae) by hot water extraction (SJ), crude polysaccharide from *Enteromorpha prolifera* (green algae) by hot water extraction (EP), crude polysaccharides from *Sargassum thunbergii* (brown algae) by hot water extraction (SJW), dilute acid extraction (STA) and dilute alkali extraction (STJ), and low molecular weight polysaccharide (SJ-D) obtained from SJ using hydrogen peroxide and ascorbic acid. Anion-exchange chromatography was performed on a DEAE-Bio Gel agarose FF (6 cm × 40 cm), eluting with water (5 L), 0.5 M NaCl (5 L) (weak binding Fraction W), 1 M NaCl (5 L) (intermediate binding Fraction I) and 2 M NaCl (5 L) (strong binding Fraction S) [6]. Two types of autohydrolyzed fractions, including higher molecular weight fraction (fraction H) and a low molecular weight fraction (fraction L), were obtained based on previous studies [7-13]. Five types of ultrafiltration devices (Ultracel 100 kDa membrane, Ultracel 50 kDa membrane, Ultracel 30 kDa membrane, Ultracel 10 kDa membrane and Ultracel 3 kDa membrane (Sigma-Aldrich) were used to separate the degraded polysaccharide mixtures prepared using 0.2 M HCOOH resulting in five fractions (fraction 100K, 50K, 30K, 10K and 3K) [6]. The desulfated polysaccharides (fraction DS) and oversulfated polysaccharides (fraction PS) were prepared based on previous studies [1, 6]. Acid degradation of polysaccharides was performed using 0.1 M HCl and 0.5 M HCl [14]. Briefly, samples were degraded by 0.1 M HCl, neutralized with 5% NH_4_OH and concentrated and precipitated with ethanol. The supernatant, as an acid-sensitive fraction, was fraction GX-1. The precipitant was further degraded with 0.5 M HCl, neutralized, concentrated and precipitated with ethanol. The supernatant was an acid-sensitive fraction, GX-2. The precipitant, as an acid-stable fraction, was GX-3. We prepared the glucuronomannan oligomers (G2, G4 and G6, corresponding to disaccharide, tetrasaccharide and hexasaccharide, respectively) and glucuronomannan polysaccharide (Gn) having a molecular weight of 7.0 k Da [15-17].

Agar, kappa-carrageenan (KC), iota-carrageenan (IC) and lambda-carrageenan (LC) were purchased from Millipore Sigma. Enoxaparin sodium injection, USP, was purchased from Teva Parenteral Medicines, Inc. Desulfated heparin (Des-H), heparosan, laminaran (LA), oxidized laminarin (LAO) and sulfated laminaran (LA-PS) were prepared as previously described [1, 18-19]. A linear sulfated fucan (SF), isolated from *Lytechinus variegates* (sea urchin), was prepared as previously described [20-21]. All samples are summarized in **Table S1**.

**Table S1** The abbreviation and structural type of polysaccharides and oligosaccharides studied.

| NO | Abbreviation | Characteristics | Ref |
| --- | --- | --- | --- |
| 1 | SJ | Fucose content (22.4%), sulfate content (30.6%), uronic acid content (10.3%), Mw (152.4 kDa), molar ratio of Man:Rha:GlcA:Glc:Gal:Xyl:Fuc (0.10:0.03:0.15:0:0.63:0.07:1) | [1] |
| 2 | SJ-W | Fucose content (15.8%), sulfate content (15.1%), uronic acid content (33.4%), Mw (77.8 kDa), molar ratio of Man:Rha:GlcA:Glc:Gal:Xyl:Fuc (0.55:0.10:0.96:0:1.22:0.37:1) | [1] |
| 3 | SJ-I | Fucose content (17.0%), sulfate content (28.3%), uronic acid content (17.4%), Mw (88.4 kDa), molar ratio of Man:Rha:GlcA:Glc:Gal:Xyl:Fuc (0.12:0.03:0.18:0:1.08:0.10:1) | [1] |
| 4 | SJ-S | Fucose content (29.0%), sulfate content (35.1%), uronic acid content (0.5%), Mw (162.7 kDa), molar ratio of Man:Rha:GlcA:Glc:Gal:Xyl:Fuc (0.02:0:0.02:0:0.27:0:1) | [1] |
| 5 | SJ-D | Fucose content (32.6%), sulfate content (37.4%), uronic acid content (6.14%), Mw (7.4 kDa), molar ratio of Man:Rha:GlcA:Glc:Gal:Xyl:Fuc (0.10:0.02:0.09:0.02:0.19:0.02:1) | [3] |
| 6 | SJ-D-W | Fucose content (27.0%), sulfate content (31.5%), uronic acid content (7.44%), Mw (6.1 kDa), molar ratio of Man:Rha:GlcA:Glc:Gal:Xyl:Fuc (0.13:0.03:0.11:0.04:0.25:0.08:1) | [3] |
| 7 | SJ-D-I | Fucose content (38.1%), sulfate content (51.1%), uronic acid content (0.6%), Mw (8.9 kDa), molar ratio of Man:Rha:GlcA:Glc:Gal:Xyl:Fuc (0.02:0:0.01:0:0.03:0:1) | [3] |
| 8 | SJ-D-S | Fucose content (41.7%), sulfate content (56.4%), uronic acid content (0.4%), Mw (9.6 kDa), molar ratio of Man:Rha:GlcA:Glc:Gal:Xyl:Fuc (0:0:0:0:0.11:0:1) | [3] |
| 9 | SJ-D-W-H | Mw (6.6 kDa), molar ratio of Man:Rha:GlcA:Glc:Gal:Xyl:Fuc (0.94:0.13:0.77:0.40:1.62:0.54:1) |  |
| 10 | SJ-D-W-L | Mw (4.3 kDa), molar ratio of Man:Rha:GlcA:Glc:Gal:Xyl:Fuc (0.37:0.07:0.87:0.07:0.40:0.14:1) |  |
| 11 | SJ-D-I-H | Mw (4.6 kDa), molar ratio of Man:Rha:GlcA:Glc:Gal:Xyl:Fuc (0.45:0.11:0.44:0.93:2.79:0.25:1) |  |
| 12 | SJ-D-I-L | Mw (2.8 kDa), molar ratio of Man:Rha:GlcA:Glc:Gal:Xyl:Fuc (0.06:0:0.11:0:0.11:0:1) |  |
| 13 | SJ-D-S-H | Mw (4.7 kDa), molar ratio of Man:Rha:GlcA:Glc:Gal:Xyl:Fuc (0.24:0:0.19:0.46:1.64:0:1) |  |
| 14 | SJ-D-S-L | Mw (3.3 kDa), molar ratio of Man:Rha:GlcA:Glc:Gal:Xyl:Fuc (0.01:0:0:0:0.04:0:1) |  |
| 15 | SJ-I-PS | Fucose content (16.1%), sulfate content (30.4%) |  |
| 16 | SJ-S-PS | Fucose content (28.4%), sulfate content (50.6%) |  |
| 17 | SJ-D-I-PS | Fucose content (37.4%), sulfate content (57.9%) |  |
| 18 | SJ-D-S-PS | Fucose content (38.6%), sulfate content (58.2%) |  |
| 19 | SJ-I-DS | Fucose content (35.8%), sulfate content (8.2%) |  |
| 20 | SJ-S-DS | Fucose content (55.8%), sulfate content (7.8%) |  |
| 21 | SJ-D-I-S | Fucose content (65.2%), sulfate content (6.2%) |  |
| 22 | SJ-D-S-DS | Fucose content (67.8%), sulfate content (6.9%) |  |
| 23 | SJ-GX-1 | Fucose content (45.5%), sulfate content (28.8%), uronic acid content (7.2%), total sugar (51.6%) |  |
| 24 | SJ-GX-2 | Fucose content (33.2%), sulfate content (21.1%), uronic acid content (14.2%), total sugar (58.4%) |  |
| 25 | SJ-GX-3 | Fucose content (6.5%), sulfate content (9.1%), uronic acid content (35.6%), total sugar (66.3%) |  |
| 26 | SJ-D-W-GX-1 | Fucose content (25.6%), sulfate content (15.9%), uronic acid content (15.6%), total sugar (56.2%) |  |
| 27 | SJ-D-W-GX-2 | Fucose content (19.8%), sulfate content (13.9%), uronic acid content (20.5%), total sugar (61.2%) |  |
| 28 | SJ-D-W-GX-3 | Fucose content (5.9%), sulfate content (8.5%), uronic acid content (39.8%), total sugar (69.8%) |  |
| 29 | G2 | β-D-glucuronosyluronic acid-(1→2)-α/β-D-mannose | [17] |
| 30 | G4 | β-D-glucuronosyluronic acid-(1→2)-α-D-mannose-(1→4)-β-D-glucuronosyluronic acid-(1→2)-α/β-D-mannose | [17] |
| 31 | G6 | β-D-glucuronosyluronic acid-(1→2)-α-D-mannose-(1→4)-β-D-glucuronosyluronic acid-(1→2)-α-D-mannose-(1→4)-β-D-glucuronosyluronic acid-(1→2)-α/β-D-mannose | [17] |
| 32 | Gn | Poly(β-D-glucuronosyluronic acid-(1→2)-α-D-mannose-(1→4), alternating) | [17] |
| 33 | STW | Fucose content (26.2%), sulfate content (18.2%), total sugar (56.7%), uronic acid content (5.7%), Mw (101.4/5.1 kDa), molar ratio of Man:Rha:GlcA:Glc:Gal:Xyl:Fuc (0.09:0:0.11:0.62:0.27:0.11:1) | [4] |
| 34 | STA | Fucose content (26.2%), sulfate content (19.3%), total sugar (68.9%), uronic acid content (8.3%), Mw (152.8/40.0/4.8 kDa), molar ratio of Man:Rha:GlcA:Glc:Gal:Xyl:Fuc (0.11:0:0.10:0.65:0.26:0.06:1) | [4] |
| 35 | STJ | Fucose content (23.8%), sulfate content (16.4%), total sugar (60.0%), uronic acid content (12.1%), Mw (151.7/6.2 kDa), molar ratio of Man:Rha:GlcA:Glc:Gal:Xyl:Fuc (0.13:0:0.08:0.25:0.28:0.12:1) | [4] |
| 36 | EP | Sulfate content (18.1%), uronic acid content (26.8%), Mw (189.3 kDa), molar ratio of Man:Rha:GlcA:Glc:Gal:Xyl:Fuc (0:1:0.37:0.13:0.06:0.31:0) | [1] |
| 37 | STW-W | Fucose content (12.3%), sulfate content (15.3%), uronic acid content (13.4%), total sugar (80.6%) |  |
| 38 | STW-I | Fucose content (30.9%), sulfate content (22.8%), uronic acid content (6.9%), total sugar (68.1%) |  |
| 39 | STW-S | Fucose content (38.6%), sulfate content (31.7%), uronic acid content (2.6%), total sugar (52.1%) |  |
| 40 | STA-W | Fucose content (20.6%), sulfate content (21.9%), uronic acid content (9.8%), total sugar (71.5%) |  |
| 41 | STA-I | Fucose content (35.6%), sulfate content (24.9%), uronic acid content (5.4%), total sugar (60.2%) |  |
| 42 | STA-S | Fucose content (41.2%), sulfate content (33.8%), uronic acid content (1.2%), total sugar (51.4%) |  |
| 43 | STJ-W | Fucose content (14.6%), sulfate content (16.8%), uronic acid content (15.6%), total sugar (78.1%) |  |
| 44 | STJ-I | Fucose content (29.5%), sulfate content (20.6%), uronic acid content (12.1%), total sugar (66.2%) |  |
| 45 | STJ-S | Fucose content (33.1%), sulfate content (25.9%), uronic acid content (5.9%), total sugar (63.8%) |  |
| 46 | EP-W | Sulfate content (8.5%), uronic acid content (12.8%), Mw (123.5 kDa), molar ratio of Man:Rha:GlcA:Glc:Gal:Xyl:Fuc (0:1:0.47:1.19:0:0.56:0) |  |
| 47 | EP-I | Sulfate content (12.2%), uronic acid content (8.8%), Mw (244.1 kDa), molar ratio of Man:Rha:GlcA:Glc:Gal:Xyl:Fuc (0:1:0.57:0.36:0.23:0.44:0) |  |
| 48 | EP-S | Sulfate content (15.5%), uronic acid content (5.8%), Mw (112.8 kDa), molar ratio of Man:Rha:GlcA:Glc:Gal:Xyl:Fuc (0:1:0.37:0:0.04:0.25:0) |  |
| 49 | Enoxaparin | Purchased from Teva Parenteral Medicines, Inc. |  |
| 50 | LC | Purchased from Millipore Sigma |  |
| 51 | IC | Purchased from Millipore Sigma |  |
| 52 | KC | Purchased from Millipore Sigma |  |
| 53 | Agar | Purchased from Millipore Sigma |  |
| 54 | Des-H | Provided by Robert J. Linhardt |  |
| 55 | Heparosan | Provided by Robert J. Linhardt | [19] |
| 56 | SF | SF, provided by Vitor H Pomin, was extracted from *Lytechinus variegates* (sea urchin). | [20-21] |
| 57 | LA-PS |  | [1] |
| 58 | LA | LA had a backbone of (1 → 3)-linked β-D-glucan, branched with (1→6)-linked β-D-Glcp and terminated with β-D-Glcp. | [18] |
| 59 | LAO | LAO had a backbone of (1→3)-linked β-D-GlcpA interspersed with (1→3, 1 →6)-linked β-D-Glcp, that was terminated with β-D-GlcpA. | [18] |

Notes: Abbreviation such as STW for crude polysaccharide from *Sargassum thunbergii* by water extraction, STA for crude polysaccharide from *Sargassum thunbergii* by dilute acid extraction, STJ for crude polysaccharide from *Sargassum thunbergii* by dilute alkaline extraction, SJ for crude polysaccharide from *Saccharia japonica* by water extraction, EP for crude polysaccharide from *Enteromorpha prolifera* by water extraction, charge fraction such as S, I , W for strong intermediate and weak, size fraction H and L for high and low, DS for desulfated; PS for oversulfated.

[1] W.H. Jin, W.J. Zhang, H.Z. Liang,Q.B. Zhang, The Structure-Activity Relationship between Marine Algae Polysaccharides and Anti-Complement Activity, Marine Drugs, 14 (2016)

[2] W. Jin, D. Jiang, W. Zhang, C. Wang, K. Xia, F. Zhang,R.J. Linhardt, Interactions of fibroblast growth factors with sulfated galactofucan from Saccharina japonica, Int J Biol Macromol, 160 (2020) 26-34.

[3] W.H. Jin, J. Wang, H. Jiang, N. Song, W.J. Zhang,Q.B. Zhang, The neuroprotective activities of heteropolysaccharides extracted from Saccharina japonica, Carbohydrate Polymers, 97 (2013) 116-120.

[4] W. Jin, W. Zhang, G. Liu, J. Yao, T. Shan, C. Sun,Q. Zhang, The structure-activity relationship between polysaccharides from Sargassum thunbergii and anti-tumor activity, International Journal of Biological Macromolecules, 105 (2017) 686-692.

[5] J. Wang, Q. Zhang, Z. Zhang, H. Song,P. Li, Potential antioxidant and anticoagulant capacity of low molecular weight fucoidan fractions extracted from Laminaria japonica, Int J Biol Macromol, 46 (2010) 6-12.

[6] W. Jin, D. Jiang, W. Zhang, C. Wang, K. Xia, F. Zhang,R.J. Linhardt, Interactions of fibroblast growth factors with sulfated galactofucan from Saccharina japonica, Int J Biol Macromol, (2020)

[7] W. Jin, W. Wu, H. Tang, B. Wei, H. Wang, J. Sun, W. Zhang,W. Zhong, Structure Analysis and Anti-Tumor and Anti-Angiogenic Activities of Sulfated Galactofucan Extracted from Sargassum thunbergii, Mar Drugs, 17 (2019)

[8] W. Jin, H. Tang, J. Zhang, B. Wei, J. Sun, W. Zhang, F. Zhang, H. Wang, R.J. Linhardt,W. Zhong, Structural analysis of a novel sulfated galacto-fuco-xylo-glucurono-mannan from Sargassum fusiforme and its anti-lung cancer activity, Int J Biol Macromol, 149 (2020) 450-458.

[9] M. Ciancia, Y. Satob, H. Nonamic, A.S. Cerezod, R. Erra-Balsellsd,M.C. Matulewicz, Autohydrolysis of a partially cyclized mu/nu-carrageenan and structural elucidation of the oligosaccharides by chemical analysis, NMR spectroscopy and UV-MALDI mass spectrometry, Arkivoc, (2005) 319-331.

[10] R.V. Menshova, S.D. Anastyuk, S.P. Ermakova, N.M. Shevchenko, V.I. Isakov,T.N. Zvyagintseva, Structure and anticancer activity in vitro of sulfated galactofucan from brown alga Alaria angusta, Carbohydrate Polymers, 132 (2015) 118-25.

[11] R.M. Rodriguez-Jasso, S.I. Mussatto, L. Pastrana, C.N. Aguilar,J.A. Teixeira, Extraction of sulfated polysaccharides by autohydrolysis of brown seaweed Fucus vesiculosus, Journal of Applied Phycology, 25 (2013) 31-39.

[12] N.M. Shevchenko, S.D. Anastyuk, R.V. Menshova, O.S. Vishchuk, V.I. Isakov, P.A. Zadorozhny, T.V. Sikorskaya,T.N. Zvyagintseva, Further studies on structure of fucoidan from brown alga Saccharina gurjanovae, Carbohydrate Polymers, 121 (2015) 207-216.

[13] S.D. Anastyuk, T.I. Imbs, N.M. Shevchenko, P.S. Dmitrenok,T.N. Zvyagintseva, ESIMS analysis of fucoidan preparations from Costaria costata, extracted from alga at different life-stages, Carbohydr Polym, 90 (2012) 993-1002.

[14] J.D. Wu, Y.J. Lv, X.X. Liu, X.L. Zhao, G.L. Jiao, W.J. Tai, P.P. Wang, X. Zhao, C. Cai,G.L. Yu, Structural Study of Sulfated Fuco-Oligosaccharide Branched Glucuronomannan from Kjellmaniella crassifolia by ESI-CID-MS/MS, Journal of Carbohydrate Chemistry, 34 (2015) 303-317.

[15] W. Jin, L. Ren, B. Liu, Q. Zhang,W. Zhong, Structural Features of Sulfated Glucuronomannan Oligosaccharides and Their Antioxidant Activity, Mar Drugs, 16 (2018)

[16] W.H. Jin, J. Wang, S.M. Ren, N. Song,Q.B. Zhang, Structural Analysis of a Heteropolysaccharide from Saccharina japonica by Electrospray Mass Spectrometry in Tandem with Collision-Induced Dissociation Tandem Mass Spectrometry (ESI-CID-MS/MS), Marine Drugs, 10 (2012) 2138-2152.

[17] W. Jin, X. He, J. Zhu, Q. Fang, B. Wei, J. Sun, W. Zhang, Z. Zhang, F. Zhang, R.J. Linhardt, H. Wang,W. Zhong, Inhibition of glucuronomannan hexamer on the proliferation of lung cancer through binding with immunoglobulin G, Carbohydrate Polymers, 248 (2020) 116785.

[18] W. Jin, X. He, W. Wu, Y. Bao, S. Wang, M. Cai, W. Zhang, C. Wang, F. Zhang, R.J. Linhardt, G. Mao,W. Zhong, Structural analysis of a glucoglucuronan derived from laminarin and the mechanisms of its anti-lung cancer activity, Int J Biol Macromol, (2020)

[19] W. Jin, S. Li, J. Chen, B. Liu, J. Li, X. Li, F. Zhang, R.J. Linhardt,W. Zhong, Increased soluble heterologous expression of a rat brain 3-O-sulfotransferase 1 - A key enzyme for heparin biosynthesis, Protein Expr Purif, 151 (2018) 23-29.

[20] V.H. Pomin, M.S. Pereira, A.P. Valente, D.M. Tollefsen, M.S. Pavão,P.A. Mourão, Selective cleavage and anticoagulant activity of a sulfated fucan: stereospecific removal of a 2-sulfate ester from the polysaccharide by mild acid hydrolysis, preparation of oligosaccharides, and heparin cofactor II-dependent anticoagulant activity, Glycobiology, 15 (2005) 369-81.

[21] I.N. Queiroz, X. Wang, J.N. Glushka, G.R. Santos, A.P. Valente, J.H. Prestegard, R.J. Woods, P.A. Mourão,V.H. Pomin, Impact of sulfation pattern on the conformation and dynamics of sulfated fucan oligosaccharides as revealed by NMR and MD, Glycobiology, 25 (2015) 535-47.

1. * Corresponding author. Tel.:+86 571 88320658; fax: +86 571 88320658.

   E-mail address: jinweihua@zjut.edu.cn

   * Corresponding author. Tel.: +518 2766839; fax: +518 2763405.

   E-mail address: zhangf2@rpi.edu

   * Corresponding author. Tel.: +518 2763404; fax: +518 2763405.

   E-mail address: linhar@rpi.edu [↑](#footnote-ref-1)
